# Accuracy of urine pH testing in a regional metabolic renal clinic: is the dipstick accurate enough?

**DOI:** 10.1007/s00240-013-0546-y

**Published:** 2013-02-09

**Authors:** Tsong Kwong, Caroline Robinson, Deborah Spencer, Oliver J. Wiseman, Fiona E. Karet Frankl

**Affiliations:** 1 Department of Urology, Royal Sussex County Hospital, Brighton, UK; 2 Department of Medical Genetics, Cambridge University, Cambridge, UK; 3 Department of Surgery, Cambridge University Hospitals NHS Foundation Trust, Cambridge, UK; 4 Division of Renal Medicine, Cambridge University Hospitals NHS Foundation Trust, Cambridge, UK; 5 Cambridge Institute for Medical Research, Addenbrooke’s Hospital Box 139, Hills Road, Cambridge, CB2 0XY UK

**Keywords:** Urine, pH, Nephrolithiasis, Clinical management

## Abstract

Urine pH is a useful marker for assessing treatment need and efficacy in patients with nephrolithiasis. Though the gold standard of measurement is with a pH electrode, dipsticks offer the convenience of cost, ease of use, and the possibility of patients measuring their own values outside the clinic. The aim of this study was to determine whether dipsticks offer the same accuracy as the electrode. Paired measurements of freshly voided urine pH with both electrode and dipstick were analysed in a multidisciplinary renal clinic. We found that although there was a high Pearson correlation between the samples (0.89, *p* = 0.001), urine dipstick measurements carried an approximately 1 in 4 risk of producing clinically significant differences (pH differences  > 0.5 pH unit) from meter values. We also found that at high and low urine pH, the dipstick tended to over- and underestimate true pH readings, respectively. Examining the values in the 98 patients where a need for pharmacological urinary pH manipulation was indicated by the true pH, we found 14 who would not have been appropriately treated, and 5 who would have been unnecessarily medicated, if the stick pH value had been used. We conclude that dipstick pH measurement is insufficiently reliable for guiding clinical decision-making.

## Introduction

Urine pH is a useful and easily measurable biochemical marker. In the context of a kidney stone clinic, it can be used to determine the need for urinary pH manipulation, and also help monitor responses to treatment.

Urine pH may be measured in various ways. In the outpatient setting, two are prevalent: dipstick testing and use of a pH meter. The latter is regarded as the gold standard [1], but is much less commonly employed than dipstick testing. A number of factors make pH meter use less attractive: first, such meters require regular calibration with test solutions; second, user training is necessary; and third, if samples are not tested when freshly voided, they must be collected under oil, which can shorten the life of the electrode. In contrast, dipsticks are single use test strips that can measure a range of variables in addition to pH, including presence of glucose, protein, leucocytes, and nitrite. They require much less user training, and with the advent of electronic readers, readings are less prone to perception bias. For accuracy, however, the need for fresh urine remains.

Dipsticks are undoubtedly convenient and are therefore in widespread clinical use for semi-quantitative detection of haematuria, proteinuria, and glycosuria. For these purposes, they are mostly sufficient for routine use, although follow-up testing by another method such as microscopy or blood testing may be required. In the urological context, accurate pH determination of a fresh urine sample is useful for proper management of the stone-forming patient. Our clinical algorithm includes alkalinizing the urine to pH ≥ 6.5 in all but calcium phosphate and struvite stone formers, where urine pH ≤ 6.5 is sought; therefore, reliable pH readings are clinically important.

The relative accuracy of dipsticks in determining urinary pH has not been determined. The aim of this study was to compare dipstick measurement of urinary pH with gold standard pH electrode readings.

## Methods

### Subjects and samples

Patients attending a regional metabolic renal clinic voided fresh urine into sterile receptacles. Each sample was immediately tested twice by a fully trained operator: once with a calibrated urine pH meter (Mettler Toledo, Leicester, UK) and again using Multistix urine dipsticks (Bayer). An electronic reader (Clinitek, Bayer) was used to assess dipstick findings. Samples were tested in random order. Patients with renal tract calcification and/or nephrolithiasis were evaluated biochemically, including 24-h quantification of urinary calcium, oxalate, urate, citrate, amino-acids, electrolytes and creatinine, and stone analysis where possible.

### Statistics

Paired dipstick and electrode measurements were correlated using the Pearson coefficient, and discrete analysis was used to further analyse the data. Absolute differences were recorded between the electrode (as standard) and dipstick (as variable) measurements. To evaluate effects on clinical management, these differences were categorised as <0.5 pH units, 0.51–1.0 pH unit, >1.01–1.5 pH units, and >1.5 units. Since the dipstick reader gives results to 0.5 units, we also measured the spread of paired meter readings associated with every 0.5 dipstick unit.

## Results

390 urine samples from 214 patients were included in the study. All patients attended the same regional metabolic renal clinic over a 4-year period. All either (1) were known to have a stone syndrome such as cystinuria; (2) were recurrent stone formers; (3) had had a single episode of nephrolithiasis under age 25; (4) had nephrocalcinosis on renal imaging; (5) had a positive family history of nephrolithiasis; or (6) had another single-gene renal disorder such as polycystic kidney disease; or a combination of these.

**Fig. 1:**
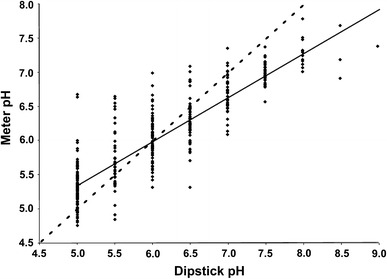
Scatter graph demonstrating range of electrode pH measurements per stated dipstick pH measurement. Line of unity is *dotted*; regression line is *solid*

Paired metered and dipstick pH readings were collected. Figure 1 illustrates the readings obtained, which statistically correlated reasonably well (Pearson Correlation Coefficient 0.89, *p* < 0.001).

Table 1 shows that the majority of readings, 290/390 or 74.4 %, were acidic (≤6.5 by meter), with the largest subgroup (116/390, 29.7 %) yielding stick values of pH 5. Examining absolute differences within pairs of measurements statistically, we initially considered pH differences of <0.5 units as clinically insignificant, since urine dipsticks only measure in steps of 0.5 units. We found that in 286 cases (73.3 %) urine pH differences were not significant, whereas in more than one in four (104/390), there was a clinically significant error. This is subcategorised as follows: 95 samples displayed apparent differences between 0.5 and 1 pH unit (24.3 %), 7 between 1 and 1.5 pH units (1.7 %), and on two occasions variation of 1.5–2 pH units was recorded (0.51 %). No sample exhibited a difference greater than 2 pH units. These data suggest that for every 4 dipstick measurements made, one would produce a clinically significant error, and that approximately one in 10 readings would give a serious error of >1 pH unit difference.

**Table 1:** pH dipstick vs. meter values

pH dipstick value	Mean (±SD) associated meter pH reading	Difference (±SD)	*n*
5	5.29 ± 0.32	0.29 ± 0.32	116
5.5	5.67 ± 0.44	0.17 ± 0.44	44
6	5.98 ± 0.34	0.01 ± 0.44	69
6.5	6.27 ± 0.32	0.23 ± 0.32	61
7	6.65 ± 0.26	0.45 ± 0.25	52
7.5	6.98 ± 0.16	0.52 ± 0.16	31
8	7.31 ± 0.22	0.69 ± 0.22	13
8.5	7.27 ± 0.4	1.23 ± 0.40	3
9	7.39	1.61	1

At stick readings of >6.5, the true pH was likely to be lower, and overall, this problem affected 220 (56.4 %) of the recordings. Of these, 63 (16.2 % of total) were >0.5 pH units. For stick pHs of 7.5 and above, all 48 (12.3 %) meter readings demonstrated this overestimate. In contrast, at lower stick pH values there was a high likelihood of underestimating the true urine pH, accounting overall for 169 (43.3 %) of the errors, of which 41 (10.5 % of total) were clinically significant. The spread of readings per dipstick category lessened as pH rose, suggesting an increase in perceived accuracy.

In 98 patients, a definite diagnosis was reached that implied the need for pH alteration, comprising one or more of hypercalciuria, calcium oxalate, urate or cystine stones, and distal RTA. Among this group, there were 19 (19.3 %) where the difference between stick and meter pHs would have led to non-adherence to the algorithm. As displayed in Table 2, the urine would not have been alkalinized had the dipstick measurement been relied upon in 14 (14.3 %) of these (i.e. the true pH was lower); in 4 (4.1 %) unnecessary alkalinization would have been commenced (i.e dipstick suggested urine pH < 6.5 whereas meter pH was ≥6.5); and in one patient (1 %), unnecessary attempts to acidify the urine would likely have been made if only stick measurements were available (Table 2). Importantly, the pH difference in these patients was not always in excess of 0.5 units, underscoring the significance of accurate measurement.

**Table 2:** Nineteen patients in whom urine pH-altering treatment would have differed without meter pH availability

Clinical indication	Stick pH	Meter pH	Difference	Consequence of stick pH-based decision
100 % Calcium oxalate stones	6.5	5.68	−0.82	Undertreatment
91 % Calcium oxalate stones	6.5	6.15	−0.35	Undertreatment
87 % Calcium oxalate stones	6.5	6.14	−0.38	Undertreatment
66 % Calcium oxalate stones	6.5	5.82	−0.68	Undertreatment
60 % Calcium oxalate stones	6.5	5.93	−0.57	Undertreatment
Hypercalciuria, calcium oxalate stone	6.5	5.80	−0.7	Undertreatment
Hypercalciuria, previous stones	6	6.99	0.99	Overtreatment
Hypercalciuria, previous stones	6.5	6.14	−0.36	Undertreatment
Hypercalciuria, previous stones	6.5	6.15	−0.35	Undertreatment
Hypercalciuria, previous stones	6.5	5.95	−0.55	Undertreatment
Hypercalciuria, previous stones	6.5	6.17	−0.33	Undertreatment
Nephrocalcinosis	5.5	6.54	1.04	Overtreatment
MSK, previous stones	6	6.57	0.57	Overtreatment
MSK, previous stones	6.5	5.98	−0.52	Undertreatment
MSK, hypercalciuria	6.5	6.15	−0.35	Undertreatment
Cystinuria	6	6.50	0.5	Overtreatment
Cystinuria	6.5	6.24	−0.26	Undertreatment
Uric acid stones	7	6.41	−0.59	Undertreatment
Calcium phosphate stones	7	6.07	−1.07	Overtreatment

*MSK* medullary sponge kidney

## Discussion

This study demonstrates that although the correlation between two different modalities of urine pH measurement is good by statistical criteria, clinically relevant discrepancies occur with an unacceptable frequency. Approximately one in four dipstick measurements yielded a clinically relevant error, the majority of these being between 0.5 and 1 pH unit from the true urine pH value. This strongly suggests that in a situation where patient management decisions are guided by the result, particularly in the patient with nephrolithiasis, dipsticks are not sufficiently accurate.

At stick pH readings <6, there was a bias towards undervaluation compared with the meter. It can be seen that although pH stick values of 6 had the closest mean electrode reading (Table 1), this was rendered less useful firstly by the wide range (Fig. 1), and secondly by the level of overlap with the true values associated with stick measurements of 5.5 and 6.5. At higher pH than this, there was a likelihood of overestimation by dipstick. Since our target pH value for treatment in all but calcium phosphate stone formers is ≥6.5, clinically unwelcome sequelae of overestimation would, as outlined above and in Table 2, have been the failure in initiation (or inappropriate withdrawal) of alkalinizing agents, or unnecessary attempts to acidify the urine of phosphate stone formers. Conversely, relying on undervalued stick pH would have led us to treat a subgroup of patients unnecessarily with urine alkalinizing agents, which many patients find unpalatable at best, and intolerable at worst. We recognize, however, that our particular clinical algorithm may not be reflected in all centres; thus, the potential management changes reported here might differ elsewhere.

pH is an inverse function of log [H^+^], and so a pH difference of only 0.1 means a 25 % increase in the concentration of H^+^ ions. Thus, a difference of 0.5 is clinically very relevant. However, although it is well recognized that the determination of urine pH is clinically useful, caveats apply. Firstly, fresh urine is required, since on exposure to air, CO_2_ will leave the urine and pH will rise. Secondly, it can be argued that dietary assessment should be added to urinalysis, since human diets are usually acidic overall, and this is borne out by our finding that almost 60 % of patients had a pH ≤ 6, a figure consistent with that found in the general population.

Our data suggest that the higher the stick pH value, the less accurate it becomes. A potential limitation of our findings is that we used the single brand of dipstick available in our clinical areas for measurement, and recent work has shown that different brands of dipstick have differing optimum pH, with some more accurate at lower pH than others [1]. Since the majority of patients that we see in the clinic appear to have acidic urine, it may be appropriate to use a dipstick that is more sensitive to lower pH levels, but this may have cost implications.

Reports of this kind are few; a study of vaginal pH measurement also found a high Pearson co-efficient with dipsticks but as with our study, wide discrepancies between the electrode reference and dipsticks [2]. In the veterinary world, a study concerning canine urinary pH again found that urine dipsticks only had moderate agreement to a variety of electrodes and could not be used for accurate measurement [3].

The relative costs of using dipsticks compared to meter measurements bear consideration, but are in fact similar. Dipsticks are about £45 + VAT for 100, and automated dipstick readers about £750. This is of the order of a laboratory grade pH meter suitable for clinical samples, plus replacement electrode and calibration fluids, the latter being a recurring cost. Clearly, proper training of health care personnel in either method is important.

The use of multisticks has already been found to confer good negative predictive value for prevention of urinary tract infections and for management of albuminuria in the community setting [4, 5]. However, positive findings had to be confirmed with laboratory testing. Urine pH is used in the clinic as a guide to therapeutic strategy, particularly in determining if alkalinization would be useful. To this end, we considered possibilities of patients treating themselves by modifying their therapy by home dipstick testing, as this might be more cost-effective than quarterly/half yearly reviews in clinic. One regime suggested by a study into bladder cancer and pH is to take two measurements, morning and evening [6]. Although in the outpatient setting we are taking “snap-shots” of the urinary pH that are dictated by the appointment time, and it is well known that urinary pH rises during the day, unfortunately our findings regarding dipsticks preclude the possibility of implementing such a strategy with confidence for stone prevention.
